# Sequence diversity of hepatitis D virus in Mongolia

**DOI:** 10.3389/fmed.2023.1108543

**Published:** 2023-03-13

**Authors:** Battur Magvan, Anne Alina Kloeble, Johannes Ptok, Daniel Hoffmann, Daniel Habermann, Anuujin Gantumur, Martha Paluschinski, Gerelmaa Enebish, Vera Balz, Johannes C. Fischer, Battogtokh Chimeddorj, Andreas Walker, Jörg Timm

**Affiliations:** ^1^Department of Microbiology and Infection Prevention and Control, Mongolian National University of Medical Sciences, Ulaanbaatar, Mongolia; ^2^Institute of Virology, University Hospital Düsseldorf, Düsseldorf, Germany; ^3^Bioinformatics and Computational Biophysics, Faculty of Biology, University of Duisburg-Essen, Essen, Germany; ^4^Institute for Transplant Diagnostics and Cell Therapeutics, University Hospital Dusseldorf, Dusseldorf, Germany; ^5^Institute of Biomedical Sciences, Mongolian National University of Medical Sciences, Ulaanbaatar, Mongolia

**Keywords:** sequence diversity, hepatitis D virus, Mongolia, HLA class I-associated selection pressure, molecular epidemiology, HDV subtypes

## Abstract

**Introduction:**

The Hepatitis Delta Virus (HDV) is a defective, single-stranded RNA virusoid encoding for a single protein, the Hepatitis Delta Antigen (HDAg), which requires the hepatitis B virus (HBV) envelope protein (HBsAg) for its transmission. Currently, hepatitis D is the most aggressive form of viral hepatitis and treatment options are limited. Worldwide 12 million people are chronically infected with HDV being at high risk for progression to cirrhosis and development of liver cancer.

**Objectives:**

Although it is well established that Mongolia is the country with the highest prevalence of HDV infections, the information on the molecular epidemiology and factors contributing to HDV sequence diversity are largely unclear. The aim of the study was to characterize the sequence diversity of HDV in rural areas from Mongolia and to determine the extent of HLA class I-associated selection pressure.

**Patients and methods:**

From the HepMongolia cohort from rural areas in Mongolia, 451 HBsAg-positive individuals were selected and anti-HDV, HDV-RNA and the sequence of the large HDAg was determined. For all individuals the HLA class I locus was genotyped. Residues under selection pressure in the presence of individual HLA class I types were identified with the recently published analysis tool HAMdetector.

**Results:**

Of 431 HBsAg positive patients, 281 were anti-HDV positive (65%), and HDV-RNA could be detected in 207 of 281 (74%) of patients. The complete large HDAg was successfully sequenced from 131 samples. Phylogenetic analysis revealed that all Mongolian HDV isolates belong to genotype 1, however, they separate into several different clusters without clear regional association. In turn, from phylogeny there is strong evidence for recent local transmission events. Importantly, we found multiple residues with strong support for HLA class I-associated selection pressure consistent with a functional CD8^+^ T cell response directed against HDV.

**Conclusion:**

HDV isolates from Mongolia are highly diverse. The molecular epidemiology suggests circulation of multiple subtypes and provides evidence for ongoing recent transmissions.

## Introduction

Hepatitis delta virus (HDV) infection causes the most severe form of viral hepatitis including faster progression toward liver-related death and hepatocellular carcinoma (1). HDV is globally prevalent, although prevalence varies greatly between countries. According to current estimates from a meta-analysis by Stockdale et al. (2020), approximately 12 million people worldwide are anti-HDV positive (2). Mongolia has the highest national anti-HDV prevalence among those testing positive for HBsAg [ranging from 35% in the global population up to 83% in high risk groups in Ulaanbaatar City (1–3)], followed by Moldova and countries in West and Central Africa (>10%) (2). Currently, the only treatment options are peginterferon alfa-2a and the entry inhibitor bulevirtide (4, 5). Unfortunately, long-term sustained HDV RNA negativity is only rarely achieved in patients treated with peginterferon alfa-2a (6, 7). Bulevirtide induces a decline in HDV RNA during treatment, however, after cessation of bulevirtide HDV RNA rapidly rebounds (6). Studies with longer treatment durations and combination of both therapies are ongoing and might result in higher cure rates (4).

HDV is a satellite virus that requires hepatitis B virus (HBV) for its virus assembly and propagation. HDV has a single-stranded, circular RNA genome approximately 1,700 nucleotides in length, that is replicated by rolling circle amplification (8). HDV virions consist of a nucleocapsid-like ribonucleoprotein (RNP), in which the HDV RNA is associated with the hepatitis D antigen (HDAg), that is enveloped by HBV surface antigen (HBsAg) (8). RNA genome editing of an amber stop codon at position 196 by a cellular adenosine deaminase (ADAR) leads to production of the large isoform with 215 aa, whereas the unedited genome produces the 196 aa isoform (9). Both isoforms undergo extensive post-translational modifications to fulfill diverse functions in genome replication, HDV RNP assembly and HDV RNP packaging by the HBsAG and inhibitors of post-translational modifications, such as lonafarnib, are currently evaluated in clinical trials (10).

HDV can be grouped into eight distinct genotypes and possibly multiple subtypes (11, 12). In a recent study, criteria for the definition of subtypes have been suggested along with a set of reference sequences for subtyping of HDV genotype 1 isolates (11). These genotypes and subtypes associate with specific global and regional distribution (3, 12, 13). The overall extent of HDV sequence diversity especially at the subtype level is not well defined. This is important information for studies of the molecular epidemiology of HDV both globally, but also locally for detection of possible transmission chains by sequence analysis. A database of HDV sequences was recently established with so far about 1,000 complete sequences of the large HDAg (14). Notably, the mechanisms driving the sequence variation of HDV have so far not been studied in detail. In recent studies, it has been shown also for HDV that CD8^+^ T cells contribute to sequence variation by selecting mutations in targeted epitopes (15, 16). HLA class I-associated selection pressure on HDV was also documented at the population level (17). Accordingly, viral sequence analysis in concert with HLA class I genotyping opens up the opportunity to detect novel epitopes under CD8^+^ T cell selection pressure. Given the sparsity of our knowledge about the targeted epitopes in HLA-diverse populations, this is highly relevant for studies in HDV immunology (18, 19).

In Mongolia, viral hepatitis is an enormous public health problem. Chronic infections with HBV or HCV are highly prevalent, each affecting between 10% and 20% of the population (3, 20, 21). Importantly, co-infections with HDV are also highly prevalent in Mongolia (3, 21, 22). This high rate of chronic hepatitis creates an immense health burden of advanced liver disease associated with liver failure and is the cause for the highest incidence rate of hepatocellular carcinoma worldwide (23–26). Despite the high number of HDV infected patients only few studies performed HDV sequence analysis (12, 27) and information on the molecular epidemiology and the extent of HDV sequence diversity of Mongolian virus isolates is not well defined (3). Furthermore, to our knowledge, there are no studies analyzing the effect of host-genetics and selection pressure on virus variability in Mongolia. To analyze the phylogenetic relationship of Mongolian HDV sequences and the influence of host-genetics on HDV sequence diversity of HDV we set up the HepMongolia cohort. For this convenience sampling cohort, people with self-reported liver disease were recruited in different areas from Mongolia. People were offered an HBsAg rapid test and individuals tested positive were included into this study.

## Materials and methods

### Patients

Samples of patients with HBV- or HBV/HDV-infection were obtained within the HepMongolia study for the surveillance of hepatitis in rural areas in Mongolia (Supplementary Table 1). Blood samples were collected from patients with self-reported liver disease from soums (small villages or small administrative regions within Mongolian provinces) in Western and Central Mongolia. A highly sensitive point-of-care-test for HBsAg (Onsite HBsAg Combo Rapid Test, #R0042C, CTK Biotech Inc., Poway, CA, United States; sensitivity 100, 95% CI [95.9%–100%]; specificity 98.3, 95% CI [95.2%–99.4%] (28)) was performed on site and HBsAg-positive patients were further studied. Written informed consent was obtained from all study participants and the study was approved by the Ethics Committee of the Mongolian Ministry of Health (study #2018-79-MEIC) and Ethics Committee of the Medical Faculty of the Heinrich Heine University Düsseldorf, Germany (#2019-404-KFogU).

### Serology

Serology was done in the routine diagnostic of the Institute of Virology Düsseldorf. HBsAg was detected and quantified with the HBsAg qualitative II Kit (#2G22-30) and the HBsAg quantitative (#6C36-43) on an Abbot ARCHITECT i2000SR (all Abbot). HDV serology was done on a Liaison-XL, (DiaSorin) using the XL Murex Anti-HDV Kit (#311260).

### Extraction of viral RNA and RT-PCR

Viral RNA from 400 μL plasma was extracted automatically using the EZ1 Virus Mini Kit v2.0 on an EZ1 Advanced XL robot or manually with the QIAamp Viral RNA Mini Kit (both Qiagen) according to the manufacturer’s protocol. RNA was eluted in a volume of 60 μL and stored at −80°C. RT-qPCR was performed with primer and probes from Mederacke et al. (29), however using the AgPath-ID One Step RT-PCR-Kit (Applied Biosystems, #4387424). To reduce hands on time, PCR mixes were prepared in large batches and frozen (“frozen-PCR mixes”). Per reaction, 25 μL RT-qPCR mixture containing 12.5 μL 2x RT-PCR-Buffer, 2.5 μL water, 1 μL of each primer (10 μM), HDV-Fwd-1 (TGGACGTKCGTCCTCCT; [positions 837 to 853]), HDV-Fwd-2 (TGGACGTCTGTCCTCCTT;[positions 837 to 854]), HDV-Rev (TCTTCGGGTCGGCATGG; [positions 891 to 907]) and 1 μL probe (10 μM) Delta-P (ATGCCCAGGTCGGAC; [positions 858 to 872]) were aliquoted in 8-strips and stored at −20°C until usage. For amplification, tubes were thawed and 5 μL of eluted viral RNA was used for RT-qPCR. Reverse transcription condition was 10 min at 45°C reverse transcription, 10 min at 95°C denaturation followed by 45 qPCR cycles each 15 s 95°C and 45 s annealing/extension at 60°C. The samples were quantified using a plasmid standard curve. The lower limit of detection was 75 copies per ml plasma and linearity was observed over the range of 1.5 × 10^3^ to 3 × 10^8^ copies/mL.

### Amplification and sequence analysis of HDV

For amplification and sequencing, a modified protocol of Karimzadeh et al. (30) was used. For reverse transcription, a “primer-mix” containing 1 μL reverse primer HDV-771R (10 μM), 1 μL dNTPs (10 mM each) and 1 μL water was aliquoted in 8-strips with hinged-caps (Eppendorf, #951010022) and stored at −20°C until usage. For reverse transcription, an appropriate number of tubes was thawed and 10 μL HDV-RNA was added to the “primer-mix.” Secondary RNA structures were melted for 5 min at 65°C and then samples were cooled down quickly to 25°C. RNA was reverse transcribed *in vitro* with Superscript III (SSIII, Invitrogen, #18080085) as previously described (31) by addition of 7 μL/well reverse transcription mix (4 μL SSIII-Buffer, 1 μL DTT, 1 μL RNase Inhibitor (NEB, #M0314L) and 1 μL SSIII) with the previously described conditions: 10 min at 25°C, 60 min at 42°C, 30 min at 50°C, 30 min 55°C, 15 min at 75°C and 4°C (31, 32). A two-step semi-nested PCR was performed with GoTaq HotStart-Polymerase (Promega, #M7401) according to the manufacturer’s protocol and the following primer combinations for PCRI: HDV-891F (AGGTCGGACCGCGAGGAGGT); HDV-339R (GCTGAAGGGGTCCTCTGGAGGTG) and PCRII: HDV-912F (GAGATGCCATGCCGACCCGAAGAG); HDV-339R (GCTGAAGGGGTCCTCTGGAGGTG). Per reaction, 95 μL PCRI mixture containing 1x GoTaq polymerase buffer, 1.5 mM MgCl_2_, 200 μM dNTPs (Bio-Budget, #80–80,015,000), 0.5 μM of each primer and 1.25 units polymerase were mixed with 5 μL HDV-cDNA. PCR condition were 120 s at 94°C, followed by 45 cycles each 30 s 95°C, 30 s 64°C and 90 s 72°C followed by 10 min at 72°C and hold at 10°C. PCRII mixes were identical to PCRI except the final volume of 97 μL. Subsequently, 3 μL of the first round PCR-product was used for the second round of nested-PCR and the annealing temperature was set to 66°C with otherwise identical PCR conditions. PCR products were purified with the QIAquick PCR-Purification Kit (Qiagen, #28106) and Sanger sequenced with sequencing primer HDV-917F, HDV-339R and HDV-1419-Seq-F (TTCTTTCCGAGAATTCCTTTGA). Sequences were submitted to Genbank and are available under accession numbers (accession numbers: OQ024240–OQ024371).

### Phylogenetic analysis of viral sequences and HAMdetector analysis

To analyze the genetic relationship and to provide the input files for the HAMdetector tool, all obtained sequences were aligned with the software Geneious 10.2.6 (RRID:SCR_010519) using MAFFT (33). For phylogenetic analysis a tree based on the large HDAg sequence, with references from Karimzadeh et al. (11), was calculated with the Mr. Bayes Plugin (34) in Geneious 10.2.8 using the GTR genetic distance model and GT3 as outgroup. For visualization the Posterior output was exported as Newick file with support values and visualized with itol (35).

HAMdetector is implemented as a julia package for identifying HLA associated substitutions based on aligned viral sequences paired to host HLA class I data. It integrates information from epitope prediction *via* MHCflurry 2.0 and phylogeny (based on RAxML-NG). The model is fit using Stan and the complete source code and documentation is available at https://github.com/HAMdetector/Escape.jl. For prediction, the large HDAg alignment used above was translated into an amino acid sequence. No adjustments were made to sequences where the amber-stop codon at position 196 was the majority. For phylogeny, the same nucleotide alignment was used as input.

### HLA class I genotyping

For HLA genotyping, an amplicon-based approach using the Illumina next generation sequencing technology (Illumina Inc.) was used. Primers were designed to target exons 2, 3, and 4 for HLA class I genes HLA-A, -B and -C, as well as class I gene HLA-DPB1, and exons 2 and 3 for HLA-DRB1 and -DQB1. Amplicons comprise the entire exon and additional intronic sequences. All primers were screened for additional SNPs using the SNPCheck software^1^ to avoid allele dropouts. Primers were purchased from Biolegio. Fragments were amplified in three multiplex PCR reactions. After clean-up using paramagnetic beads, sample-specific barcodes and Illumina compatible adapter sequences were added by PCR. Samples were pooled, underwent an additional purification step and were quantified using the QuantiFluor dsDNA system (Promega, # E2670). Seven pM of the NGS library were applied to the MiSeq instrument (Illumina Inc.) for a paired-end 2 × 280 cycles run using a standard v3 cartridge according to the manufacturer’s instructions. As an internal quality run control, we used a spike-in of 15% PhiX. After de-multiplexing of the samples by the MiSeq Reporter software (Illumina Inc.), the analysis of the read sequences was performed by a self-developed software (NGSAnalyser) taking into account quality control values and high coverage to automate data analysis. Algorithms were developed to distinguish between sequencing artifacts such as cross-over products and closely related alleles. The American Society for Histocompatibilty and Immunogenetics (ASHI) approved the entire NGS workflow including the self-developed software NGSAnalyser. The Institute of Transplantation Diagnostics and Cell Therapeutics (ITZ), University Hospital of Düsseldorf, Düsseldorf, is accredited to perform HLA typing for routine diagnostic purposes.

## Results

### Phylogenetic analysis of HDV isolates from Mongolia shows high sequence diversity and evidence for local transmission

**Figure 1:**
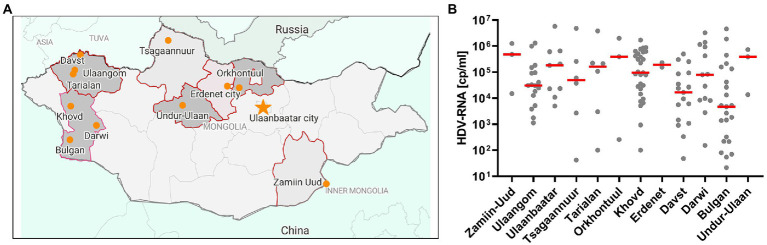
Recruitment of the HepMongolia cohort. **(A)** Sampling regions for the HepMongolia cohort. The map was created with data from open street map and visualized with datawrapper. **(B)** HDV viral load in the different sampling regions.

**Figure 2:**
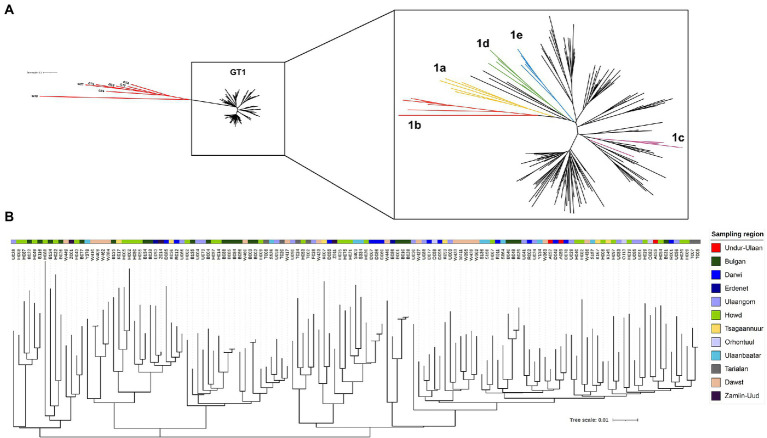
Phylogenetic analysis of viral sequences covering the large HDAg. A total of 131 HDV sequences covering the large HDAg were aligned with reference sequences [from (11)] using MAFFT. A phylogenetic tree was calculated with the Mr. Bayes Plugin (34) in Geneious 10.2.8 using the GTR genetic distance model and GT3 as outgroup. **(A)** *Left:* unrooted tree all sequences from the HepMongolia cohort together with reference sequences with assigned genotypes. Genotypes 2–8 are colored red, GT1 samples in black. *Right:* Inset showing only GT1 samples. Sequences from the HepMongolia cohort are colored black, GT1 subtypes described by [(11)] are color coded. **(B)** Disperse geographical distribution of HDV GT1 subtypes in Mongolia. The color code is according to the sampling region.

In an approach to identify patients with HBV-infection in rural areas from Mongolia, individuals with self-reported liver disease were screened for HBsAg with a lateral-flow point-of-care test in 11 soums in 11 different provinces between 400 and 1,400 km West of Ulaanbaatar and one soum in the South East (Figure 1A). A total of 431 individuals with detectable HBsAg were identified (Table 1) of which 281 (65%) tested positive for anti-HDV (range 46%–73%). Of the 281 anti-HDV positive individuals, 207 (74%) had detectable HDV RNA in serum. The HDV RNA concentrations are shown in Figure 1B. The complete large HDAg (L-HDAg) was successfully sequenced from 131 individuals. In line with previous publications (27), phylogenetic analysis confirmed that all isolates were HDV genotype 1 (Figure 2A). Importantly, within the genotype 1 clade, sequences from Mongolia formed distinct phylogenetic clusters consistent with multiple subtypes. In a recent study, criteria for HDV subtyping were proposed and the authors suggested a reference set of sequences with assigned subtyping (11). These reference sequences were included in the phylogenetic analysis with the Mongolian sequences with the reference sequences color-coded for the five different subtypes 1a to 1e (Figure 2A). Notably, sequences from Mongolia clustered with subtypes 1c and 1d, however, the tree suggests further differentiation of isolates from Mongolia into so far unassigned subtypes. There are at least three additional highly reproducible clusters supported by bootstrap values >75%. As the samples were collected from different regions of Mongolia, we tested if the clusters associated with specific regions where samples were obtained (Figure 2B). However, samples collected from the same district were distributed across the whole tree and did not associate with specific clusters, strongly suggesting that regional transmission is not a major driver for these clusters. Interestingly, despite this overall high sequence diversity, there were also examples where two or three isolates have a particularly small genetic distance, indicating a common transmission event. Of note, in all these putative individual transmission events, samples were collected from the same soum consistent with local transmission. Taken together, there is overall high sequence diversity between HDV isolates collected in rural areas from Mongolia. The molecular epidemiology overall suggests regionally independent transmission of HDV, although on a local level there are individual examples strongly supporting a direct transmission event.

**Table 1:** HDV prevalence among the adult population of rural areas of Mongolia.

Province	Region	Size of settlements	HBsAg positive (*n*)	Female sex HBsAg positive *n* (%)	Median age HBsAg positive *n* (%)	Anti-HDV positive *n* (% of HBsAg)	HDV-RNA positive *n* (% of anti-HDV positive)	Female sex HDV-RNA positive *n* (%)	Median age HDV-positive
Arkhangai province	Undur-Ulaan	Soum (village)	13	6 (46)	49 (26–64)	6 (46)	4 (66)	2 (50)	53 (52–64)
Khovd province	Bulgan	Soum (village)	70	35 (49)	48 (15–86)	51 (73)	36 (71)	17 (47)	45 (21–64)
Khovd province	Darwi	Soum (village)	48	32 (64)	44,5 (16–61)	28 (58)	23 (82)	14 (60)	51 (37–61)
Uvs province	Davst	Soum (village)	51	21 (41)	46 (21–75)	37 (73)	24 (65)	10 (41)	49 (28–64)
Erdenet	Erdenet	City	15	5 (31)	32,5 (27–59)	10 (67)	7 (70)	2 (28)	33,5 (28–39)
Khovd province	Khovd	Provincial center	70	45 (64)	45 (23–70)	48 (69)	40 (83)	28 (70)	46 (25–62)
Selenge province	Orkhontuul	Soum (village)	18	12 (66)	42 (28–58)	11 (61)	7 (64)	3 (42)	52 (37–56)
Uvs province	Tarialan	Soum (village)	13	10 (76)	45 (28–69)	9 (69)	8 (89)	6 (75)	37 (32–60)
Khuvsgul province	Tsagaannuur	Soum (village)	18	8 (44)	43,5 (27–62)	12 (67)	9 (75)	5 (55)	40,5 (31–55)
Ulaanbaatar	Ulaanbaatar	Capital city	46	22 (47)	40,5 (32–58)	25 (54)	14 (56)	8 (57)	41 (33–58)
Uvs province	Ulaangom	Provincial center	62	38 (61)	46,5 (24–76)	40 (65)	31 (78)	20 (64)	50 (32–71)
Dornogovi province	Zamiin-Uud	Soum (village)	7	4 (57)	34 (26–48)	4 (57)	4 (100)	3 (75)	34 (32–44)
	Total		431	238 (55)	44 (18–86)	281 (65)	207 (74)	118 (56)	47 (21–71)

### HLA-class I-associated selection pressure on the large HDAg contributes to the sequence diversity of HDV in Mongolia

Next, we analyzed if amino acid substitutions in the large HDAg are under positive selection in the presence of distinct HLA-molecules. It was previously described that HDV evades from the CD8^+^ T cell response by selecting mutations in targeted epitopes (15, 17, 36, 37). These studies utilized cohorts from Europe or North America for analyses of CD8^+^ T cell responses and the possible consequences of immune escape of HDV. We therefore addressed if similar mechanisms contribute to the HDV sequence diversity in Mongolia and whether the epitopes under selection pressure are shared across populations. We used the recently published tool HAMdetector (38) to probabilistically quantify the strengths and uncertainties of associations between HLA class I alleles and amino acid substitutions. The tool integrates several pieces of information that could contribute to an explanation of apparent HLA-substitution associations in a single coherent Bayesian regression model, with strong associations probably being the consequence of HLA class I selection pressure. The integrated pieces of information include: alignment of viral sequences, HLA alleles of patients, sparsity of HLA substitution associations, phylogeny of viral sequences, and possible HLA class I-binding motifs. The resulting posterior probabilities of HLA substitution associations lie between 0 and 1 and can be interpreted easily. For instance, a posterior probability of 0 speaks strongly against an association, a value of 0.5 neither favors nor disfavors an association, and a value of 1 strongly supports an association and, hence, an HLA-selected mutation.

**Figure 3:**
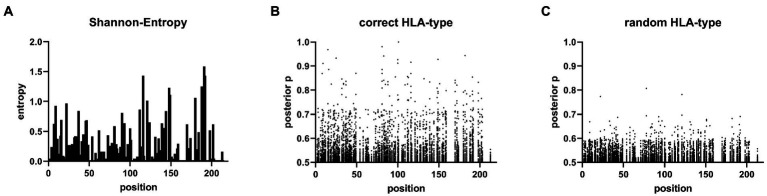
Sites under selection predicted by Bayesian regression model. **(A)** Shannon entropy map of the 131 L-HDAg Sequences. **(B)** Posterior probability values >0.5 of the Bayesian regression model from the HAMdetector for each HLA-molecule on every amino acid in the L-HDAg are shown. **(C)** Posterior probability values of an HAMdetector run with the same alignment however with randomly assigned HLA-type.

Viral sequence data and HLA class I genotypes were available for 131 individuals from the HepMongolia cohort. The HLA distribution of the HepMongolia cohort (Table 2) was comparable to previous studies from Mongolia (39–41). Notably, the extent of sequence variation at the amino acid level was not evenly distributed across the large HDAg. The Shannon entropy as a measure for the amino acid diversity at individual positions strongly varied including highly diverse as well as conserved residues (Figure 3A). This suggests that that different areas of the HDAg are subject to varying degrees of positive selection (e.g., by immune pressure) or negative selection (e.g., due to functional constrains).

**Table 2:** Comparison of the HLA polymorphism of the Mongolian population.

	HepMongolia (*n* = 131;2022)	Mongolia Buryat (*n* = 141; 2002)	Mongolia Khalkha (*n* = 200;2002)	Mongolia Khalkha pop 2 (*n* = 202;1995)	Mongolia Oold (*n* = 104; 2002)	Mongolia Tarialan Khoton (*n* = 85;1996)	Mongolia Tsaatan (*n* = 144;2002)	Mongolia Ulaanbaatar Khalkha (*n* = 41;1996)
HLA-A*01	0.084	0.114	0.080	0.075	0.144	0.066	0.125	0.085
HLA-A*02	0.302	0.501	0.265	0.279	0.327	0.094	0.243	0.192
HLA-A*03	0.046	0.036	0.060	0.030	0.058	0.079	0.069	0.038
HLA-A*11	0.122	0.075	0.100	0.106	0.077	0.107	0.076	0.069
HLA-A*23	0.015	0.015	0.015	0.005	0.039	0.000	0.021	0.012
HLA-A*24	0.183	0.246	0.195	0.208	0.183	0.214	0.222	0.231
HLA-A*26	0.046	0.014	0.060	0.067	0.058	0.094	0.028	0.049
HLA-A*29	0.023	0.008	0.010	0.010	0.010	0.048	0.000	0.000
HLA-A*30	0.015	0.011	0.020	0.021	0.000	0.082	0.021	0.037
HLA-A*31	0.057	0.083	0.085	0.070	0.058	0.065	0.049	0.049
HLA-A*32	0.008	0.046	0.020	0.010	0.000	nd	0.021	nd
HLA-A*33	0.065	0.004	0.060	0.050	0.039	0.000	0.125	0.089
HLA-A*68	0.034	0.029	0.015	0.015	0.000	0.000	0.000	0.026
HLA-B*07	0.042	nd	0.055	0.025	0.067	nd	0.034	nd
HLA-B*08	0.031	nd	0.035	0.052	0.010	nd	0.014	nd
HLA-B*13	0.069	nd	0.040	0.042	0.039	0.041	0.056	0.049
HLA-B*15	0.092	nd	0.115	0.057	0.067	0.036	0.111	0.077
HLA-B*18	0.008	nd	0.005	0.011	0.010	0.041	0.000	0.000
HLA-B*27	0.015	nd	0.025	0.010	0.058	0.006	0.021	0.000
HLA-B*35	0.069	nd	0.075	0.065	0.096	0.079	0.014	0.024
HLA-B*37	0.073	nd	0.035	0.042	0.029	0.018	0.069	0.066
HLA-B*38	0.015	nd	0.020	0.005	0.010	0.128	0.014	0.024
HLA-B*39	0.008	nd	0.010	0.010	0.000	0.006	0.000	0.000
HLA-B*40	0.103	nd	0.145	0.206	0.183	nd	0.174	nd
HLA-B*41	0.008	nd	0.000	nd	0.010	nd	0.014	nd
HLA-B*44	0.050	nd	0.040	0.030	0.048	0.094	0.028	0.049
HLA-B*46	0.019	nd	0.005	0.005	0.019	0.000	0.000	0.012
HLA-B*48	0.076	nd	0.035	0.057	0.039	0.035	0.021	0.073
HLA-B*50	0.034	nd	0.020	0.035	0.010	nd	0.035	nd
HLA-B*51	0.084	nd	0.125	0.080	0.125	0.067	0.083	0.148
HLA-B*52	0.034	nd	0.025	0.020	0.039	0.071	0.049	0.037
HLA-B*54	0.038	nd	0.050	0.055	0.039	0.024	0.021	0.012
HLA-B*55	0.038	nd	0.025	0.015	0.019	nd	0.042	nd
HLA-B*56	0.015	nd	nd	0.020	nd	nd	nd	nd
HLA-B*57	0.015	nd	0.020	0.098	0.000	0.029	0.007	0.024
HLA-B*58	0.050	nd	0.070	nd	0.077	0.055	0.194	0.110
HLA-B*73	0.007633588	nd	0.005	nd	0	nd	0	nd

HLA-B*49 and HLA-B*67 had each only 1 participant and were therefore excluded.

Figure 3C shows the results of the analysis when HLA class I genotypes were randomly assigned to the viral sequences and serves as a control with no associations being expected. Only posterior probabilities > 0.5 are illustrated to focus on possible evidence for HLA class I-associated mutations. The majority of the posterior probabilities are below 0.7 with only few exceptions (Figure 3C; Supplementary Table 2). In contrast, when the true HLA class I genotypes were assigned to the viral sequences (Figure 3B), multiple substitutions with high posterior probabilities were detected. These substitutions are indicative for highly reproducible HLA class I-associated selection pressure on the L-HDAg in this cohort (Supplementary Table 3). In a previous study, there was experimental support for targeted CD8^+^ T cell epitopes in HDV when posterior probabilities were above 0.8 (38). We therefore also used this cut-off to compare the results from the HepMongolia cohort to HDV isolates from Europe (38). According to these criteria, a total of 45 HLA class I-associated mutations were detected in 31 different positions of the large HDAg from Mongolia (Table 3). Interestingly, in only four positions the same or similar HLA-associated mutations were previously detected in a European cohort of patients with HDV infection suggesting that only some HAMs are shared across HLA-diverse populations. Two of these four shared HAMs were located inside previously described CD8^+^ T cell epitopes restricted by the relevant HLA class I type. Of note, two additional HAMs were also located inside previously described CD8^+^ T cell epitopes, however, here, no evidence for selection was detected in the European cohort.

**Table 3:** List of HAMs predicated on posterior probabilities.

HepMongolia				Habermann et al.			
Post.prob.	Allele	Position	Substitution	Substitution	Post. Prob.	Exp. confirmed	Comments
1	B*37	101	E	D101E	0.96	100-Q**D**HRRRKAL-109	Karimzadeh et al. (2019); see B*37 position 100
0.98075	A*68	81	I	V81I	0.97		
0.968	A*33	15	D	-	-		
0.9435	B*51	182	H	-	-		
0.942	A*33	83	A	-	-		
0.93275	B*48	25	R	-	-		
0.92775	C*07	149	T	-	-		
0.9265	B*37	100	E	-	-	100-**Q**DHRRRKAL-109	Karimzadeh et al. (2019); see B*37 position 101
0.9155	B*40	116	T	-	-		
0.912	A*01	100	E	-	-		
0.9105	A*33	9	K	-	-		
0.88575	B*54	81	I	-	-		
0.8855	A*33	16	I	-	-		
0.86975	B*54	49	L	-	-		
0.86925	A*33	81	I	-	-		
0.869	C*07	8	K	-	-		
0.86875	B*50	116	N	-	-		
0.8565	B*48	112	K	-	-		
0.85475	A*33	97	E	-	-		
0.854	B*58	191	G	-	-	189-RG**S**QGFPW-196	Kefalakes (2019)
0.852	B*08	113	N	K113N	0.96		
0.847	C*04	132	K	-	-		
0.846	B*13	32	K	-	-		
0.84075	C*04	170	D	-	-		
0.8405	A*33	83	T	-	-		
0.83675	B*58	81	I	-	-		
0.8365	C*07	37	T	-	-		
0.83475	A*33	88	K	-	-		
0.83375	B*27	139	G	-	-		
0.83225	B*54	198	L	-	-		
0.82975	B*15	170	D	S170N	0.99	170-**S**MQGVPESPF-179	Karimzadeh et al. (2019)
0.82775	A*31	34	T	-	-			
0.82625	B*13	151	D	-	-			
0.825	C*07	112	K	-	-			
0.82475	A*33	19	E	-	-			
0.8245	B*51	34	V	-	-			
0.8235	B*51	85	S	-	-			
0.822	B*51	88	G	-	-			
0.82125	A*03	37	T	-	-			
0.82125	B*40	109	Q	-	-			
0.82025	A*68	172	R	-	-			
0.816	B*08	116	R	-	-			
0.80875	B*13	81	V	-	-			
0.808	B*54	148	S	-	-			
0.80725	B*40	32	R	-	-			

**Figure 4:**
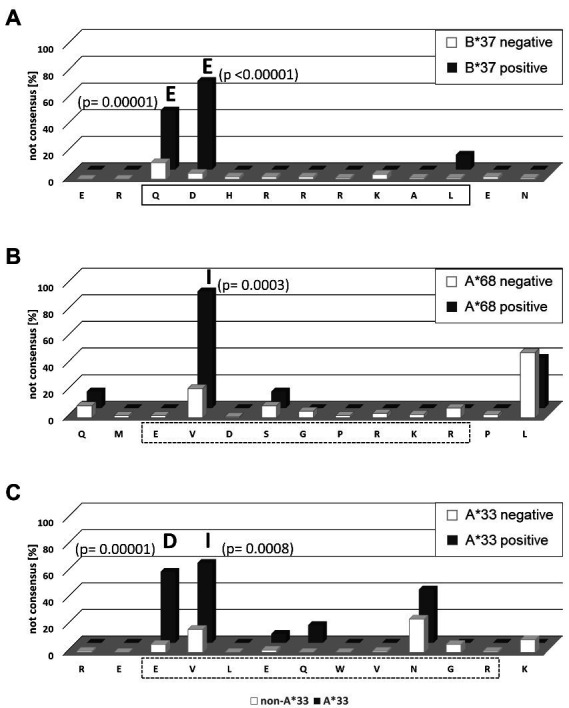
Frequency of HLA-associated viral polymorphisms in the epitope region predicted by the HAMDetector. The frequency of variations from the prototype sequence of the epitope region are shown for patients carrying the corresponding HLA-allele (black) and patients not carrying the corresponding HLA-allele (white). Positions with significant differences in polymorphism frequencies in the absence or presence of the corresponding HLA-molecule are marked, and *p* values (Fisher exact test) and the most frequent variant amino acid are indicated. **(A)** HLA-B*37 epitope region aa98–110. The confirmed epitope is outlined. **(B)** HLA-A*68 epitope region aa78–90. The predicted epitope is outlined in dashed lines. **(C)** HLA-A*33 epitope region aa13–25. The predicted epitope is outlined in dashed lines.

The shared HAMs between both cohorts included the two substitutions with the highest posterior probability indicating selection pressure in the presence of HLA-B*37 and HLA-A*68 (Table 3). The exact frequencies of viral sequence polymorphisms in the HAM-containing region in the presence and absence of the relevant HLA class molecule are shown in Figure 4. In the previously described HLA-B*37-restricted epitope (QDHRRRKAL), substitutions are highly enriched at position 1 and 2 of the epitope (positions 100 and 101 of the L-HDAg) in the presence of HLA-B*37 but not in the absence, consistent with reproducible selection pressure in HLA-B*37-positive individuals (Figure 4A). In the context of HLA-A*68, substitutions were highly enriched at position 81 of the large HDAg (Figure 4B). Although in the same position substitutions were also enriched in a European cohort, no CD8^+^ T cell epitope has yet been confirmed. Notably, the HAM is located at position 2 of a peptide sequence that is predicted to bind to HLA-A*68 (Figure 4B). Importantly, the majority of HAMs in the Mongolian cohort are novel and may hint at novel, previously undetected epitopes. Figure 4C shows the region 13–25 of the large HDAg where the substitutions E15D and V16I are highly enriched in HLA-A*33 positive individuals. This region also encodes for a peptide sequence predicted to bind with high affinity to HLA-A*33 consistent with a novel epitope under selection pressure in Mongolia. Taken together, there is strong evidence that HLA-class I-associated selection pressure contributes to the sequence diversity of HDV in Mongolia.

## Discussion

Mongolia is the country with the highest prevalence of viral hepatitis. The prevalence of HBsAg in the population is about 10%–12% with a decreasing trend in younger cohorts since the introduction of hepatitis B vaccination programs in 1991 (3). The reported percentage of anti-HDV positive individuals in Mongolian HBsAg carriers varies greatly with reported frequencies of 35% in the general population up to 83% in specific risk groups (2, 3, 21, 27). In our study, the proportion of anti-HDV positive individuals in HBsAg carriers was 65%. When recruiting patients for the study, persons with self-reported liver disease were targeted, which may explain an increased prevalence of HBV/HDV co-infection compare to the general population. HDV RNA was detected in 74% of the anti-HDV positive patients, which is in line with previous studies in populations with high anti-HDV prevalence (1, 42). Notably, the median age of people with HDV infection in this cohort was 47 years, with 30% of them being younger than 40 years suggesting still a high disease burden in younger cohorts. The main objective of the present study was to better characterize the sequence variability of HDV in Mongolia. For this purpose, the complete genomic region of large HDAg was successfully sequenced from 131 individuals. In the remaining HDV RNA-positive samples, sequencing was incomplete or unsuccessful, in most cases due to a relatively low viral load in the sample.

In the phylogenetic analysis, all isolates were assigned to genotype 1. In previous studies of HDV genotypes in Mongolia, genotype 1 was also the most common (2, 21, 27). Criteria for exact definitions of subtypes have not been established for HDV to date. Recently, criteria for subtyping HDV genotype 1 samples have been proposed and a dataset of reference sequences for the subtypes was published (11). Although some of the isolates from Mongolia clustered with reference sequences, no clear assignment to a subtype was possible for most isolates. With regard to the described subtypes, the isolates from Mongolia were most likely to be assigned to subtypes 1c and 1d, with subtype 1c dominating. Thus far, subtype 1c was mainly found in isolates from East Asia such as China, Vietnam or Japan, but also seems to occur frequently in Mongolia. Some isolates were most likely to be assigned to subtype 1d, which was so far described predominantly in Turkey and Iran. Interestingly, in the phylogenetic analysis, further clusters were formed that cannot be clearly assigned to any of the described subtypes.

Analysis of the phylogeny of the viral sequences opened up the opportunity to study the molecular epidemiology of HDV in Mongolia. Importantly, there was no clear evidence for a regional clustering of the viral sequences. The sequences from samples collected in the same regions from Mongolia distributed across the whole tree. This is in line with the traditionally high mobility of people living in Mongolia. Especially in rural areas people are typically sedentary only for short periods of time. It is not well established when HDV was introduced and started to spread through the Mongolian population. The differentiation of HDV sequences from Mongolia in clusters may suggest that founder effects by introduction and spreading of different isolates contributed to the molecular epidemiology. However, additional studies in larger datasets will be required to address this in more detail.

Despite the lack of evidence for regional clustering of HDV isolates, there was strong evidence for local transmission events. We identified four examples with two or three isolates showing a remarkably low genetic distance, suggesting either direct transmission or a shared recent transmission event. Notably, in all four cases the respective isolates were collected at the same site, implying possible direct contacts. Unfortunately, no additional information on the contact networks or possible transmission risk factors are available for this cohort. Nevertheless, identification of such putative transmission events indicates that sequence analysis of HDV isolates can strongly contribute to a better understanding of the epidemiology (43) and may improve the characterization of the relevant infection routes (44). Moreover, when the putative transmissions can be placed in a temporal context, the analyses may allow conclusions about the incidence of HDV infections (45). We believe, the sequence in this study data support that recent transmission events still occur in Mongolia.

We provide evidence that the high HDV sequence diversity observed in Mongolia is at least partly caused by positive selection. Notably, the study does not directly address the extent of negative selection on the large HDAg. Negative selection refers to the process by which certain genetic traits are eliminated from a population because they confer a disadvantage to replication (46). As the Shannon entropy was not evenly distributed and some amino acids were highly conserved, negative selection appears to influence the genetic plasticity of HDV. This is in line with functional constrains of the multi-functional HDAg (47). In turn, our analysis with the HAMdetector tool indicates that HDV is under strong positive selection by HLA class I-associated selection pressure, which contributes to the HDV sequence diversity in Mongolia. Adaptation to CD8^+^ T cell immune pressure by selection of escape mutations is well established for different viruses (48–50). Also, for HDV there is evidence supporting the selection of escape mutations from viral sequencing studies at the population level as well as from studies of individual CD8^+^ T cell responses (15–17, 30, 51). The selected mutations typically interfere with the interaction of an infected cell with the T cell, either by impairing presentation of the variant epitope or by altering binding of the variant HLA/peptide-complex to the T cell receptor (48). The full epitope repertoire that is targeted in the L-HDAg in the context of different HLA-types is not well defined yet and epitope mapping was just recently started (15, 17, 36, 37). Lack of experimental confirmation of novel epitope candidates is an important limitation of this study, however, isolation and storage of PBMCs for functional studies was not possible and would require a much larger effort and more advanced infrastructures. Nevertheless, the dataset provided here can support ongoing epitope mapping efforts by pointing toward regions under CD8^+^ T cell selection pressure. A few residues with support for HLA class I-associated selection pressure were located inside described epitopes or overlapped with HLA-associated mutations from a previous study of a European cohort (17, 38). However, the majority of the associations were unique in the Mongolian cohort and did not overlap with associations in the European cohort. This highlights that viruses may differentially adapt in HLA-diverse populations as it has been suggested for HCV or HIV (52–54). Although this was not formally addressed in this study, such differential selection processes between populations may contribute to the formation of clusters or subtypes. Adaptation of HDV at the population level to common HLA molecules is an additional challenge for the development of T cell based immunotherapies, when escape variants are selected and accumulate.

Taken together, we provide a large dataset of HDV sequences from Mongolia in order to describe the extent of sequence diversity. Our data confirm previous studies showing that genotype 1 is the most frequent. Nonetheless, within this genotype the isolates from Mongolia belong to different clusters or to novel subtypes that have not yet been characterized. Phylogenetic analysis support that recent local transmission still occurs in Mongolia. One important driving factor for HDV sequence diversity is the adaptation to HLA class I-associated selection pressure.

## Data availability statement

The datasets presented in this study can be found in online repositories. The names of the repository/repositories and accession number(s) can be found at: GenBank, OQ024240–OQ024371.

## Ethics statement

The studies involving human participants were reviewed and approved by Ethics Committee of the Mongolian Ministry of Health (study #2018-79-MEIC) and the Ethics Committee of the Medical Faculty of the Heinrich Heine University Düsseldorf (#2019-404-KFogU). The patients/participants provided their written informed consent to participate in this study.

## Author contributions

This project was conceived by AW, BC and JT. Mongolian ethical permission, sample collection and sample logistic was coordinated by BC. Samples were collected by GE, AG, BM and BC. Experiments were performed by BM and AK. Data were analyzed by all authors. The manuscript was written by AW, MP and JT with input from all authors. All authors contributed to the article and approved the submitted version.

## Funding

This study was funded by grants from the *Deutsche Forschungsgemeinschaft* (TI 323/4-1), the *Stiftung zur Erforschung infektiös-immunologischer Erkrankungen* (AW, 10-16-72), the Jürgen Manchot Foundation and the German Foreign Exchange Service (DAAD) PAGEL (project nos. 54448058 and 57220593).

## Conflict of interest

The authors declare that the research was conducted in the absence of any commercial or financial relationships that could be construed as a potential conflict of interest.

## Publisher’s note

All claims expressed in this article are solely those of the authors and do not necessarily represent those of their affiliated organizations, or those of the publisher, the editors and the reviewers. Any product that may be evaluated in this article, or claim that may be made by its manufacturer, is not guaranteed or endorsed by the publisher.

## Footnotes

^1^ https://genetools.org/SNPCheck/snpcheck.htm
